# Clinical Factors Influencing End-of-Life Care in a Chinese Pediatric Intensive Care Unit: A Retrospective, *post-hoc* Study

**DOI:** 10.3389/fped.2021.601782

**Published:** 2021-04-07

**Authors:** Yueniu Zhu, Xiaodong Zhu, Lili Xu, Mengyan Deng

**Affiliations:** Department of Pediatric Critical Care Medicine, Xinhua Hospital, Affiliated to Shanghai Jiaotong University School of Medicine, Shanghai, China

**Keywords:** clinical decision-making, pediatric intensive care units, withdrawing care, withholding treatment, withdrawing treatment, end-of-life care

## Abstract

**Objective:** End-of-life(EOL) care decision-making for infants and children is a painful experience. The study aimed to explore the clinical factors influencing the EOL care to withhold/withdraw life-sustaining treatment (WLST) in Chinese pediatric intensive care unit (PICU).

**Methods:** A 14-year retrospective study (2006–2019) for pediatric patients who died in PICU was conducted. Based on the mode of death, patients were classified into WLST group (death after WLST) and fCPR group (death after full intervention, including cardiopulmonary resuscitation). Intergroup differences in the epidemiological and clinical factors were determined.

**Results:** There were 715 patients enrolled in this study. Of these patients, 442 (61.8%) died after WLST and 273 (38.2%) died after fCPR. Patients with previous hospitalizations or those who had been transferred from other hospitals more frequently chose WLST than fCPR (both *P* < 0.01), and the mean PICU stay duration was significantly longer in the WLST group (*P* < 0.05). WLST patients were more frequently complicated with chronic underlying disease, especially tumor (*P* < 0.01). Sepsis, diarrhea, and cardiac attack (all *P* < 0.05) were more frequent causes of death in the fCPR group, whereas tumor as a direct cause of death was more frequently seen in the WLST group. Logistic regression analysis demonstrated that previous hospitalization and underlying diseases diagnosed before admission were strongly associated with EOL care with WLST decision (OR: 1.6; *P* < 0.05 and OR: 1.6; *P* < 0.01, respectively).

**Conclusions:** Pediatric patients with previous hospitalization and underlying diseases diagnosed before admission were associated with the EOL care to WLST.

## Introduction

Most post-neonatal pediatric intensive care unit (PICU) admissions account for majority of pediatric deaths in developed countries, especially in the United States (1, 2). However, recent technological advances in pediatric research have significantly reduced the mortality rates among PICU admissions by prolonging the life of critically ill patients (3–5). Three recent multicenter studies have reported a decline in PICU mortality rates of ~<3% in western countries (6–8). There is no official data of mortality in Chinese PICU.

Nowadays, little attention has been paid to end-of-life (EOL) care, following the ineffectiveness of therapeutic interventions. In collaborative decision-making concerning EOL care for their children, parents are given complete freedom to decide, especially in case of medical futility (9, 10). As a result, the ethical practices of withholding and withdrawing life-sustaining treatments (WLST) in children (11), have become common in PICUs in many countries (2, 12, 13). Consequently, the goals of care get redefined—to ensure support and comfort to the patient's parents rather than providing a cure, and to attend to the psychosocial needs of dying children and their families which will promote optimal EOL care, and thus facilitate a peaceful death (14, 15). Major factors have been shown to strongly influence the decision to WLST including, geographic parameters, religious and cultural beliefs, societal values, race, legal concerns, subjective evaluation of the benefits and burdens of life support, the nature of the underlying disease, the quality of life prior to hospitalization, and the expected suffering secondary to the pathology (12, 16, 17). Thus, the incidence of deaths after WLST vary worldwide (12). Several earlier studies have identified high variability in the prevalence of WLST in the ICU setting (15). Of note, substantial variability exists in WLST across world regions and countries, among individual ICUs within a country, and among individual patients within a single ICU. Moreover, the mean prevalence of deaths after WLST in patients varied from 0 to 84.1% between different studies (SD: 23.7%) whereas the mean prevalence of deaths after withholding also varied from 5.3 to 67.3% (mean: 27.3%, SD: 18.5%) (12). Earlier, studies have reported that ~28–65% of all PICU deaths follow a restriction in care in North America and Europe (11, 18). On the contrary, WLST is less common in South America and some Asian countries, and WLST cases account for ~18–50% of ICU deaths (19, 20).

Although there have been a couple of studies elucidating EOL care in Japanese PICUs (20, 21), to the best of our knowledge, there are no studies evaluating the characteristic factors associated with WLST in EOL care in Chinese PICUs. Therefore, in view of the above, we aimed to document the epidemiologic and clinical characteristics that influence the EOL care to WLST of PICU patients at one of the largest tertiary referral hospitals in China.

## Methods

### Study Design

This study was performed at Xinhua Hospital, Shanghai, which is one of the largest general hospital with many pediatric subspecialties in China, and is also a tertiary referral center for children with complex pediatric conditions. About 1,000–2,000 pediatric patients were admitted to the PICU every year. The PICU is a comprehensive intensive care facility engaged in the full spectrum of pediatric critical care, including medical/surgical, cardiac and trauma care. This is a 14-year retrospective study that involved *post-hoc* analysis of data from our previously published study (22). Pediatric patients (*n* = 715) from 1 month to 11 years of age who died in the PICU between 2006 and 2019 were included.

The study protocol was approved by the Ethics Committees of Xinhua Hospital, Shanghai (XHEC-D-2020-0110), and the need for written informed consent was waived by the committee as all data were used retrospectively and de-identified.

### Data Collection

All data used in this study were drawn from the databases of the hospital information services department. These databases prospectively recorded demographic and clinical data of all hospitalized children. The children who died in the emergency department were not included due to incomplete medical records. Data collected included diagnoses, demographic characteristics, hospital units caring for patients, symptoms, diagnosis on PICU admission, source of PICU admission, occurrence and timing of EOL decision-making, mode of death, time and place of death, and information of patients' family members. The immediate cause of death refers to the diagnosis that predominantly and directly played a role in the pathophysiology leading to the occurrence of a life-threatening situation. An underlying chronic disease refers to any complicated chronic disease diagnosed before death. They included congenital abnormalities, immunodeficiency and/or autoimmune diseases, tumors, and other disorders, such as malnutrition, obesity, and hemophilia.

### Modes of Death

Since the 1990s, WLST practices have been well-defined in literature (14, 23). We examined WLST status at death based on medical records. All deaths were classified into two groups: WLST group (death after WLST) (14) and fCPR group [death after full intervention, including cardiopulmonary resuscitation (CPR)]. Withdrawal of life-sustaining treatment was defined as discontinuing the life-sustaining intervention/treatment that was already in place. Withholding treatment was defined as the non-initiation or the decision not to escalate a life-sustaining treatment (14, 24, 25). Life-sustaining treatments included CPR, inotropic or vasoactive medication, mechanical ventilation, renal replacement therapy, and extracorporeal membrane oxygenation (ECMO).

These two decisions were made by the parents following the evaluation of patients' prognosis and/or potential quality of life after surviving a critical episode. A poor prognosis indicated an expected poor quality of life after the critical episode. Each patient was evaluated individually by a medical team (including the nurse) together with the family. In this evaluation, the presence of chronic underlying diseases, the prior quality of life, and the expected suffering secondary to the sequelae/pathology were considered. The patient's family was informed in written that the patient was in a life-threaten situation and that death would be the possible outcome. All decisions concerning the WLST status were made during the patients' PICU stay. For families that opted for WLST, Statement of Consent forms required by the standard institutional protocol were signed either by the parents /guardians prior to the procedure.

### Statistical Analyses

All analyses were performed using GraphPad Prism (version 8 for Mac OS, GraphPad Software, La Jolla California USA, www.graphpad.com). Categorical data were expressed in terms of frequency and percentage, and Chi-square test was performed for comparison. Nonparametric continuous variables were expressed as median with interquartile range (IQR). For continuous data, the Mann-Whitney *U*-test was performed to compare variables between two groups. After conducting these bivariate analyses comparing the patient characteristics and clinical factors in WLST group and fCPR group, we then used a multiple logistic regression analysis to identify the factors that influencing EOL care. The dependent variable was EOL care as WLST. The independent variables were those showed *p* < 0.05 in the bivariate analysis. A *P*-value of < 0.05 was considered statistically significant.

## Results

### Basic Characteristics

During the 14-year study period, there were a total of 715 (3.6%) patients died among the 19, 813 patients admitted to the PICU. Of these 715 patients, 442 (61.8%) died after WLST and 273 (38.2%) died after fCPR. Approximately 59.6% of pediatric patients who died were male, while 40.4% were female. The median age was 19 months (IQR: 5–54 months). In this study, 72% of the deaths occurred in children below 5 years of age, of whom 40.7% were infants. Children aged 5–11 years accounted for about 28% of the deaths (Table 1).

**Table 1:** Characteristics of all patients.

**Characteristics**	***N* = 715**
Age, median (IQR)	mo (5–54mo)
1–12 months, No. (%)	291 (40.70)
1–4 years, No. (%)	225 (31.47)
5–11 years, No. (%)	199 (27.83)
**Gender, No. (%)**	
Male	426 (59.58)
Female	289 (40.42)
**End-of-life decisions, No. (%)**	
fCPR	273 (38.18)
WLST	442 (61.82)
**Source of admission to PICU, No. (%)**	
Other hospitals	431 (60.28)
ER/Outpatient departments	195 (27.27)
Previous hospitalizations, No. (%)	569 (79.58)
With chronic underlying diseases, No. (%)	559 (78.18)
Congenital abnormalities	291 (40.70)
Tumors	193 (27.00)
Immunodeficiency/autoimmune diseases	69 (9.65)
**Immediate cause of death, No. (%)**	
Pneumonia	233 (32.59)
Sepsis	108 (15.10)
CNS infections	44 (6.15)
Diarrhea	19 (2.66)
Non-traumatic intracranial/gastrointestinal hemorrhages	71 (9.93)
Cardiac shock	78 (10.91)
Tumors	114 (15.94)
Accidents	41 (5.73)
Duration of Hospital stay (days), median (IQR)	7 (3,17)
Duration of PICU stay (days), median (IQR)	5 (2,11)

*fCPR, failed cardiopulmonary resuscitation; WLST, withhold/withdraw life-sustaining treatment; PICU, pediatric intensive care unit; ER, emergency room; CNS, central nervous system; IQR, interquartile range*.

### Clinical Factors Associated With WLST and fCPR

Notably, 79.6% (*n* = 569) of patients had a history of previous hospitalization, whereas 60.3% (*n* = 431) were transferred from other hospitals before admission to the PICU (Table 2). The proportion of patients who were transferred from other hospitals and those with previous hospitalizations were significantly higher in the WLST group than the fCPR group (*P* = 0.0009 and *P* < 0.0001, respectively). The lengths of hospital and PICU stays of patients in the WLST group were longer than those in the fCPR group (*P* = 0.0527; *P* = 0.0132, respectively).

**Table 2:** Clinical factors influencing end-of-life decisions.

**Characteristics**	**fCPR (*n*/273)**	**WLST (*n*/442)**	***P*-value**
Age, median (IQR)	18 (5–60)	20 (5–53)	0.6866^a^
Male, No. (%)	172 (60.30)	254 (57.47)	0.1427^b^
Source of admission, No. (%)			
Other hospitals	143 (52.38)	288 (65.16)	0.0009^b^
ER/Outpatient departments	84 (30.77)	111 (25.11)	0.1014^b^
Previous hospitalizations, No. (%)	192 (70.33)	377 (85.29)	<0.0001^b^
Duration of Hospital stay (days), median (IQR)	6 (2,16)	8 (3,17)	0.0527^a^
Duration of PICU stay (days), median (IQR)	4 (2,11)	5 (2,12)	0.0132^a^

a Mann-Whitney test;

b *Chi-square test*.

*P < 0.05 is statistically significant. fCPR, failed cardiopulmonary resuscitation; WLST, withhold/withdraw life-sustaining treatment, PICU, pediatric intensive care unit; ER, emergency room; IQR, interquartile range*.

### Chronic Underlying Diseases for Different EOL Care

The distribution pattern of chronic underlying diseases for different EOL care was shown in Table 3. Approximately 78.2% of patients presented with chronic underlying diseases, including congenital abnormalities (40.7%), tumors (27%), and immunodeficiency/autoimmune diseases (9.7%). The proportion of patients who had chronic underlying diseases was significantly higher in the WLST group than in the fCPR group (68.1 vs. 52.8%). The proportion of patients pre-existing chronic diseases diagnosed before PICU admission was also significantly higher in the WLST group than in the fCPR group (68.1 vs. 52.8%,). Pediatric patients in the WLST group were more likely to have tumors than patients in the fCPR group (31.9 vs. 19.1%). Although the occurrence of congenital abnormalities was more frequent than the other occurrence of chronic underlying diseases in all patients, no significant differences were observed between the two EOL care groups.

**Table 3:** Chronic underlying diseases for different end-of-life decisions.

**Characteristics, No. (%)**	**fCPR (*n*/273)**	**WLST (*n*/442)**	***P*-value^a^**
With Chronic underlying diseases	193 (70.70)	366 (82.81)	0.0002
Congenital abnormalities	107 (39.19)	184 (41.63)	0.5317
Tumors	52 (19.05)	141 (31.90)	0.0002
Immunodeficiency/Autoimmune diseases	31 (11.36)	38 (8.60)	0.2418
Underlying diseases diagnosed before PICU admission	144 (52.75)	301 (68.10)	<0.0001

a *Chi-square tests*.

*P < 0.05 is statistically significant. fCPR, failed cardiopulmonary resuscitation; WLST, withhold/withdraw life-sustaining treatment; PICU, pediatric intensive care unit*.

### Immediate Causes of Death for Different EOL Care

The distribution of immediate causes of death for different mode of death was shown in Table 4. Infectious diseases caused the majority of the deaths in children. Patients with fCPR decision were more like to have a diagnosis of immediate cause of death as sepsis, diarrhea and cardiac attack, compared with those in the WLST group (*P* < 0.05). On the contrary, the patients in the WLST group were more likely to have a diagnosis of tumor as the direct cause of death when compared with those in the fCPR group (*P* < 0.0001).

**Table 4:** Immediate causes of death for different end-of-life decisions.

**Immediate cause of death, No. (%)**	**fCPR (*n*/273)**	**WLST (*n*/442)**	***P*-value^a^**
Infectious diseases	160 (58.61)	244 (55.20)	0.3935
Pneumonia	78 (28.57)	155 (35.07)	0.0845
Sepsis	52 (19.05)	56 (12.67)	0.0239
CNS infections	18 (6.6)	26 (5.9)	0.7495
Diarrhea	12 (4.4)	7 (1.5)	0.0303
Non-infectious diseases	113 (41.39)	198 (44.80)	0.3935
Non-traumatic intracranial/gastrointestinal hemorrhages	24 (8.8)	47 (10.63)	0.4434
Cardiac shock	46 (16.85)	32 (7.24)	0.0001
Tumors	22 (8.06)	92 (20.82)	<0.0001
Accidents	17 (6.23)	24 (5.43)	0.7409

a *Chi-square test*.

*P < 0.05 is statistically significant. fCPR, failed cardiopulmonary resuscitation; WLST, withhold/withdraw life-sustaining treatment; PICU, pediatric intensive care unit, CNS, central nervous system*.

### Multivariable Analysis of Factors Associated With EOL Care

In multivariable analysis, the history of previous hospitalization and the underlying diseases diagnosed before PICU admission significantly associated with WLST decision (OR: 1.599; and OR: 1.598; respectively, Table 5). The source of admission as transferred from other hospitals tended to be an independent factor influencing the WLST decision; however, this did not reach statistical significance (*P* = 0.06). We selected the independent variables showing significant difference (*p* < 0.05) in binary analysis (Tables 2–4) for the univariable and multivariable logistic regression test. In the multiple logistic regression analysis, age and gender were remained as covariates. With chronic underlying diseases was eliminated from the model since it was related with underlying diseases diagnosed before PICU admission, while latter showed significant association with EOL care.

**Table 5:** Logistic regression of factors associated with end-of-life care.

**Factor**	**Univariate**		**Multivariate**	
	**OR (95%CI)**	***P*-value**	**OR (95%CI)**	***P*-value**
Age	0.998 (0.995–1.002)	0.4667	0.998 (0.994–1.002)	0.3410
Male	0.793 (0.581–1.080)	0.1430	0.767 (0.557–1.053)	0.1026
Transferred from other hospital	1.700 (1.250–2.314)	0.0007	1.404 (0.985–1.999)	0.0600
Previous hospitalization	2.447 (1.693–3.550)	<0.0001	1.599 (1.001–2.556)	0.0493
Underlying diseased diagnosed before PICU admission	1.932 (1.417–2.639)	<0.0001	1.598 (1.117–2.285)	0.0101
Duration of PICU stay	1.003 (0.998–1.011)	0.3917	1.002 (0.997–1.009)	0.5505

*P < 0.05 is statistically significant. OR, odds ratio; CI, confidence interval; PICU, pediatric intensive care unit*.

## Discussion

Our 14-year retrospective observational study described the differences of epidemiologic and clinical characteristics of the pediatric patients with the different decisions of WLST in the PICU in one of the largest tertiary referral hospital in China. The present study showed that WLST decision was commonly associated with PICU deaths and was more frequent in patients with previous hospitalization and underlying diseases diagnosed before admission. Our findings provided a better understanding of factors influencing EOL care decisions, which could help support the decision-making for these family and health care professionals.

As highlighted previously, we found that PICU deaths accounted for most deaths within our tertiary care pediatric hospital (14). A previous study in a Japanese PICU reported the mortality rate to be ~3.3%, which was consistent with that reported in our study (3.6%). In contrast, the mortality rate reported in Western countries have been shown to be <3% (6–8). This difference could be attributed to the differences in geographic parameters, disease distribution, race, and medical systems, which could in turn influence the modes of death among or within countries (19, 26, 27).

Recent prospective, multicenter studies have reported that approximate 70 and 30% of patients died in a PICU setting following WLST and fCPR/brain death, respectively (28). Studies have also showed that a majority of deaths (60–85%) were due to the WLST (8, 26, 29). Similarly, our study found that ~62% and 38.2% of deaths occurred after WLST and fCPR, respectively. Our study findings are also in line with other recent reports from PICUs worldwide (30–32). Over the past decade, WLST decisions have become relatively acceptable in critically ill patients in China, but there is very few research in the this field in China yet.

Similar to a previous study, this study demonstrated that the incidence of deaths in patients with previous hospitalization history and that of patients who were transferred from other hospitals were significantly higher in the WLST group than in the fCPR group (33). Our study also suggested that previous history of hospitalization tend to be an independent factor of influencing WLST decision and source of admission as transferred from another hospital before PICU admission was strongly associated with WLST. These findings indicated that after children being diagnosed at another hospital, the parents of these critical-ill children sought a second opinion and/or alternate hospital to understand the severity and impact of the child's underlying condition before acknowledging the futility of further therapeutic efforts. Therefore, such families, after reaching a consensus with the healthcare professionals involved in their child's care, were more likely to choose WLST as the EOL care for their child.

The present study found that underlying chronic diseases diagnosed before PICU admission tend to be an independent factor of WLST decision. Our results were further corroborated by previous studies that showed that nearly two-thirds of all deaths in PICUs are attributable to chronic or pre-existing diagnoses (2, 31). Cancer is a known predictor for WLST (34). Moreover, parents of patients diagnosed with tumor were aware of the clinical outcome. Therefore, parents of such patients are more likely to choose WLST when treatment efforts fail to avoid prolonged suffering. The lengths of hospital and PICU stays were longer in the WLST group than in the fCPR group, similar to previously reported results (35, 36).

Our study showed that the incidence of infectious diseases was significantly higher than that of non-infectious diseases, suggesting that infectious diseases are the leading cause of death in most PICU patients. Similar to previous reports, our study demonstrated that the most common immediate causes of deaths in hospitalized children were pneumonia, tumors, sepsis, cardiac shock, non-traumatic intracranial and gastrointestinal hemorrhage (37). Although pneumonia was the most common immediate cause of death, it did not influence the EOL decision. Furthermore, the incidences of sepsis, diarrhea, and cardiac shock were significantly more common in the fCPR group than in the WLST group. In contrast, tumors were more commonly observed in the WLST group (21%) than in the fCPR group. The most plausible explanation for this could be that sepsis, diarrhea, and cardiac attack were acute diseases and often present atypical symptoms at the early stages but the conditions could deteriorate dramatically as the disease progresses. In such cases, if the circumstances of cardiac or respiratory arrest were envisaged, the family and the physician would be more willing to believe that the disease was reversible, which in turn might impede the acceptance of EOL decision until fCPR.

There are several limitations that warrant discussion. First, this study was retrospective and only included deaths that occurred at a tertiary health care facility. Second, we did not examine several patient characteristics known to influence EOL care, including family history, religion, culture, race, and socioeconomic status due to lack of data availability in some patients. The descriptive nature of the study could not fundamentally identify the causal relationship between the observed characteristics and outcomes. Further investigations should be carried out to explore the relationship between a patient's family background and EOL decision. Third, the children who died in the emergency department were not included due to incomplete medical records. Then, this was a single-center study. Thus, our study may not reflect the general practice of PICUs in China. The results of the study should been interpreted bearing in mind that they came from the practice of East Asia. Nevertheless, this study will be the first step forward to reveal and improve EOL care in Chinese PICUs.

## Conclusions

The present study showed that WLST was a common practice in EOL care in a Chinese PICU. EOL care of WLST were frequent in patients transferred from other hospitals before admission to PICU, with longer lengths PICU stay and suffered from chronic disease as caner. Patients with fCPR were more common to be diagnosed with sepsis, diarrhea, and cardiac shock as the immediate cause of death. Patients with previous hospitalization and underlying diseases diagnosed before admission were strongly associated with WLST decision for EOL care. A better understanding of these factors associated with decisions to WLST is critical to physicians, policy makers, ethicists, and healthcare leaders to provide optimal EOL care for pediatric patients and improved the quality of life for families.

## Data Availability Statement

The original contributions presented in the study are included in the article/supplementary material, further inquiries can be directed to the corresponding author/s.

## Ethics Statement

The studies involving human participants were reviewed and approved by Ethics Committees of Xinhua Hospital, Shanghai. Written informed consent from the participants' legal guardian/next of kin was not required to participate in this study in accordance with the national legislation and the institutional requirements.

## Author Contributions

YZ is responsible for the design of this study, patient data analyses, and manuscript preparation. XZ is responsible for the design of this study and interpretation of the results. LX and MD contributed to data collection from the hospital database. MD reviewed the data. All authors contributed to the article and approved the submitted version.

## Conflict of Interest

The authors declare that the research was conducted in the absence of any commercial or financial relationships that could be construed as a potential conflict of interest.
